# Accelerating the Adoption of eSource in Clinical Research: A Transcelerate Point of View

**DOI:** 10.1007/s43441-020-00138-y

**Published:** 2020-03-03

**Authors:** Abhijit A. Parab, Prasann Mehta, Arundhati Vattikola, Christine K. Denney, Michele Cherry, Rakesh M. Maniar, Jesper Kjaer

**Affiliations:** 1 Global Clinical Data Management and Central Monitoring, Bristol-Myers Squibb, Route 206 & Province Line Road, Princeton, NJ 08543 USA; 2 Global Data Management & Standards, Merck & Company Inc., 2000 Galloping Hill Road, Kenilworth, NJ 07033 USA; 3 Business Technology Services, One Health Plaza, East Hanover, Novartis, NJ 07936 USA; 4 Medicines Development IT, Eli Lilly and Company, Lilly Corporate Center, Indianapolis, IN 46285 USA; 5 Business Technology, Pfizer Inc., 235 East 42nd Street, New York, NY 10017 USA; 6 R&D IT, Novo Nordisk A/S, Vandtårnsvej 114, 2860 Søborg, Denmark

**Keywords:** TransCelerate, eSource, Direct data capture (DDC), Electronic health record (EHR), Applications, Devices

## Abstract

For almost a decade, regulators and pharmaceutical industry groups have been interested in electronic source (eSource) in clinical trials (Nordo et al. in Learn Health Syst 3:e10076, 2019). eSource may provide efficiencies and value; however, eSource adoption is fragmented and slow. Acceleration of eSource adoption is a critical step in modernizing the conduct of clinical trials. The desired future state is one in which all source data, acquired through any context (e.g., healthcare delivery, chronic disease management) and actor (e.g., healthcare professional, patient, caregiver), are completely electronic, adequate in quality, and fully acceptable in clinical trial submissions by regulators worldwide. Achieving this desired future state requires transformative change management to foster adoption and minimize the burden of implementing eSource. Realizing this vision requires collaborative and dedicated efforts from multiple stakeholders, including patients, clinical trial participants, sites, technology vendors, standards organizations, regulators, payers, and sponsors. Stakeholders should align upon guidance to promote data integrity, data privacy, data security, and interoperability. The eSource revolution requires open dialogue, inclusive of shared learnings among stakeholders, to collectively and rapidly advance adoption. Adoption of eSource will optimize clinical research by enabling faster access to research data and more rapid decision-making, increasing clinical trial efficiency. Furthermore, adoption of eSource will improve data integrity by allowing direct data flow from the source to the sponsor’s system, with minimal or no human intervention. This paper provides the TransCelerate point of view (POV) and recommendations to achieve the future state vision of complete utilization of eSource data in clinical trials and builds on previous TransCelerate eSource publications.

## Introduction

Since 2010, the [1] European Medicines Agency (EMA), United States (US) Food and Drug Administration (FDA), United Kingdom’s Medicines and Healthcare products Regulatory Agency (MHRA), and Japan’s Pharmaceuticals and Medical Devices Agency (PMDA) have all either expressed interest in or provided written guidance on their expectations regarding clinical source data in electronic form (eSource) [2–6]. Likewise, industry groups such as the Society for Clinical Data Management (SCDM), Clinical Data Interchange Standards Consortium (CDISC), and eClinical Forum (eCF) have commented on eSource from varying perspectives [7–13].

There is consensus among these groups that the use of eSource in clinical trials benefits multiple stakeholders [14–16]. eSource in clinical trials may:

- Improve protocol design and clinical trial participant recruitment [17],
- Improve, modernize, and streamline data collection [16], monitoring, [18] and reporting,
- Improve access to electronic health data to advance/enable machine learning for healthcare,
- Enhance the site and clinical trial participant experience,
- Reduce data entry errors and minimize the effort required for source data verification,
- Facilitate risk-based monitoring (RBM),
- Promote real-time access for data review [4, 19],
- Enable more rapid identification of safety and operational signals [21], as well as increase data integrity [7, 20] and quality [7, 14, 18, 22], and
- Demonstrate the value of drugs/therapy for outcome-based evidence generation [23]

TransCelerate’s two landscape papers [14, 15] (available at https://www.transceleratebiopharmainc.com/esource-assets/) categorize eSource into four (4) distinct types:

1. **Electronic health records (EHR)** The collection and reuse of site/patient electronic health record system data for use in clinical research.
2. **Devices & Apps** The collection and management of clinical data from non-site personnel (e.g., clinical trial participants and caregivers) using mobile devices, including smartphone or tablet applications (e.g., electronic clinical outcome assessment), wearables, and sensors (e.g., glucose monitor, smart pill, remote chemistry, and ambient sensors).
3. **Non-case report forms (non-CRFs)** The collection and transfer of data in electronic format from internal sponsor sources (e.g., specialty laboratories) or external vendors (e.g., laboratory results, imaging, electrocardiograms [ECGs], randomization, drug accountability) into clinical research data repositories/warehouses without entering the data into an electronic Case Report Form (eCRF).
4. **Direct data capture (DDC)** The direct entry of clinical data by site staff into a mobile application or electronic data capture (EDC) system.

While the TransCelerate Sponsor Landscape paper [14] defines the distinct types of eSource, this POV paper describes the changes necessary to accelerate all types of eSource implementations and move towards a future state in which the global use and acceptance of eSource is fully achieved.

## Future State

Facilitating the global implementation of eSource to scale is a daunting challenge. It will require a change in mindset from what is currently considered “source data” (i.e., a mixed model of paper and electronic source data recorded at the point of generation) to a future state that considers only electronic data as source data, when data are initially recorded in electronic form.

The future state vision includes the following:

- Engaging patients and sites during the clinical trial design phase,
- Delivering improved and timely patient outcomes through monitoring adherence and demonstrated efficacy,
- Promoting end-to-end data integrity and secure data collection, aligned with established regulatory expectations for privacy, validation, and control,
- Solving interoperability constraints through effective piloting, application of standards, and leveraging innovative technology platforms, and
- Enabling multi-modal data collection in clinical trials, allowing clinical trial participant data to be collected from various sources.

This desired future state will only be possible through transformative change management designed to minimize the burden on those implementing new eSource technologies and processes.

Achieving this future state will require a global, cross-industry, collaborative effort built on engagement across all stakeholders who:

- Conduct research (e.g., clinical trial participants, sites, academia),
- Provide systems, devices, sensors, and apps (e.g., vendors of EHR, DDC, and electronic Clinical Outcomes Assessment [eCOA]),
- Create standards (e.g., Clinical Data Interchange Standards Consortium [CDISC], Health Level Seven International [HL7]),
- Regulate drug development and protect public health (e.g., global health authorities, public health centers, Centers for Disease Control and Prevention [CDC], Institutional Review Boards [IRBs], Ethical Review Boards [ERBs]),
- Sponsor research (e.g., pharmaceutical companies, academia, government), and
- Pay for outcome-based treatment (e.g., private health insurer, government).

Stakeholders should:

- Collaborate with global regulatory agencies to align and establish harmonized eSource implementation guidelines,
- Optimize established and/or maturing eSource modalities,
- Further develop nascent eSource technologies (e.g., DDC, devices, sensors, apps, EHR), and
- Advance healthcare data and research data interoperability & reusability.

The broad implementation of eSource will enable modernization of the clinical trial to include end-to-end digital data flow (i.e., from eSource data collection to clinical data platform analysis).

## Roadmap

Our desired future state builds on the established data collection principles already in use today, while addressing future needs and nuances in implementation. We recognize that the path to achieving end-to-end eSource implementation varies by sponsor because each sponsor company has its own unique set of eSource implementation challenges to solve. In addition, there is currently no ‘one-size-fits-all’ solution when it comes to eSource implementation approaches, nor are there any available harmonized regulatory eSource adoption guidelines from regulatory agencies, such as the FDA, MHRA, European Medicines Agency (EMA), PMDA, and Health Canada (HC). The differences are attributed to the following:

- Country-specific or region-specific regulatory requirements,
- Clinical trial phase and complexity of clinical trial design, and
- Therapeutic area of interest.

eSource implementations pose challenges in five key areas:

I. Clinical trial design, protocol, and data collection,
II. Automated data exchange, security, and privacy,
III. New roles,
IV. Regulatory alignment, and
V. Collaboration

For each key area, we describe the current mitigation effort or proposed solutions for associated challenges. Most, if not all, solutions require sponsor, site, clinical trial participant, vendor, regulator, and payer collaboration to move towards the future state vision and provide value to all stakeholders.

## Key Area I: Clinical Trial Design, Protocol, and Data Collection

**Figure 1.**
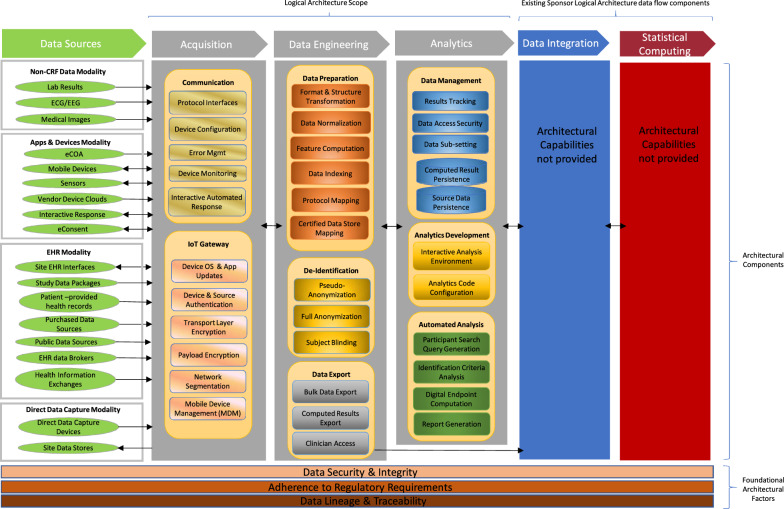
eSource Logical Architecture.

Optimizing eSource implementation starts with clinical trial design and digitizing the protocol. Clinical trial designs should consider the eSource strategy and proactively define the eSource content (e.g., clinical trial-specific metadata [see Data Lineage & Traceability in Fig. 1]) to be automatically collected during clinical trial execution via all data collection systems and supporting business processes [24–26]. Utilizing eSource data collection during the clinical trial would enable automated data collection, promote and facilitate remote monitoring, and improve access and traceability of the collected data.

The TransCelerate eSource Logical Architecture describes the components of the end-to-end flow. This type of transformative architecture will allow near real-time data access and review, improved clinical trial participant safety monitoring, quicker clinical trial decision-making, and a more collaborative approach to clinical trial execution across sponsors, investigators, regulators, and payers (i.e., outcome-based payments trials) while positively impacting clinical trial participant safety, improving data integrity [20, 38], and improving overall clinical trial conduct [22].

The clinical trial design process should also include patients and site engagement to provide valuable insights during protocol design and digitization, ensuring reduced burden on these stakeholders. For greater clinical trial participant engagement, digital technologies used during clinical trial execution could be integrated into the clinical trial design to enable remote participation, where permitted by local laws, regulations, and ethics committees.

### Challenges

#### Lack of Appropriate Stakeholder Involvement in Clinical Trial Design

*The appropriate people involved in protocol and Clinical Study Report (CSR) development are not involved in the clinical trial design, which results in re-work*.

*Proposed solution* Identify the potential eSource modalities in the protocol and include eSource subject matter experts (SMEs) in the clinical trial design. eSource SME acumen should broadly cover the functions and the systems shown in the logical architecture diagram (see Fig. 1). During clinical trial design, stakeholders can provide varying perspectives on the clinical trial design (e.g., patients, sites, technology vendors, regulators). Having a full set of resources defined and consulted will ensure that the right people with the right skillsets are involved.

*Value* eSource modalities included in the protocol may reduce delays in clinical trial execution. Having the correct resources defined and consulted will reduce the number of protocol amendments and reduce the delays in execution.

#### Access to and Corrections of Source Data

*Guidance and processes for querying and correcting source data are unclear*.

*Proposed solution* Apply consistent guidance and efficient processes for data corrections. Correction of data must occur in the permanent recording of the data; however, there is no clear global definition of data source. Guidance and processes are necessary to define the true data source and instruct organizations on data correction processes for central labs, EHR, and DDC. We recommend the following:

- For EHR, the true data source is the point of generation and corrections are performed within the source that feeds the EHR.
- For apps, devices, and DDC, the true data source lies within the third-party server; however, the corrections could be performed through the corresponding eSource modality or on the third-party servers.
- For non-CRF sources, the true data source lies within the third-party server and the corrections are performed within the third-party servers.

*Value* Clear guidance and processes would assist organizations in adhering to existing International Council for Harmonization of Technical Requirements for Pharmaceuticals for Human Use (ICH) requirements.

#### Site and Clinical Trial Participant Burden

*Current eSource implementation approaches may increase the burden on sites and clinical trial participants (e.g., data entry in EDC required even when eSource is used, lack of clinical trial participant and site perspective on the adoption of eSource options, lack of training for the site and clinical trial participants)*.

*Proposed solution* Consider appropriate input from sites and patients in protocol design and workflow creation. The use of eSource should not increase the clinical trial participant or site burden; rather, it should provide a positive clinical trial experience from an operational perspective. During clinical trial design, sponsors should include site and patient perspectives on the use of the specific eSource technology being considered in the clinical trial. Sponsors should engage with patient advocacy groups and site advocacy groups to get feedback on eSource technology. Sponsors may gain additional insight into the usability of the workflow by involving sites and patients in workflow usability testing (e.g., eCOA device workflow usability). Sponsors should also consider improvements to the current process, training, and technologies to support eSource adoption.

*Value* Improved patient and site engagement will ultimately lead to successful adoption of eSource with a positive user experience and accelerate the drug development process. In addition, sponsors may benefit from improved internal business processes.

#### Technological Challenges

*Use of technology causes unexpected behaviors and/or errors at point of data capture*.

*Proposed solution* Develop risk assessments by modality to mitigate technology issues. Risk assessments should be completed by modality to understand how the technology operates and simulate what could happen under a variety of circumstances. To reduce impact from eSource modality failures, sponsors should perform risk mitigation on an ongoing basis and ensure that business continuity plans (BCPs) are in place.

*Value* Performing due diligence may result in reduced risk to clinical trial participants and increased acceptability of data, allowing timely submission of a marketing application for a new drug fulfilling an unmet medical need.

## Key Area II: Automated Data Exchange, Security, and Privacy

In the future state, seamless standards, technology, and processes should exist for easy, direct, and secure global eSource data exchanges (incorporating data lineage/traceability and privacy aspects) across multiple organizations and at scale. Clinical data will be transferred automatically system-to-system and device-to-system using secure networks or using a Standard API (Advanced Programming Interface) (see Data Sources, Acquisition, Data Security & Integrity, and Adherence to Regulatory Requirements in Fig. 1).

A global data broker, such as the Health Information Exchanges (HIE) in the United States [27] and the European Union (EU) Innovative Medicines Initiative (IMI) Electronic Health Records Systems for Clinical Research (EHR4CR) [28], or European Health Data & Evidence Network (EHDEN) [29] platforms, could facilitate the automated data exchanges via an agreed-upon industry data mapping strategy or data standards. A broker-facilitated, secure clinical data exchange between the site EHR, the site clinical research database (e.g., DDC system, if applicable), and the sponsor (e.g., EDC, data warehouse) could enable a many-to-many, integrated, scalable model versus today’s single-site point-to-point, file-based approaches [7, 28]. Early-use cases for a data broker model, as described in the IMI EHR4CR platform [28], are for protocol feasibility assessments and facilitating site recruitment. The future cases may utilize HL7′s Fast Healthcare Interoperability Resources (FHIR) to focus on pre-populating clinical research CRFs with EHR data, complemented with EDC data [30]. Challenges with integration and scalability may more rapidly overcome through adoption of electronic health record exchange formats at the governmental level [31].

Integrating EHRs and a sponsor platform, such as an EDC or data warehouse/eSource platform, would bring efficiencies to both site and sponsor workflows by eliminating duplicate data entry and data reconciliation between the source and sponsor database. With emerging trends on adoption of eSource (e.g., EHR and DDC), the current landscape will likely transform away from an EDC-centric clinical trial database design. Future-use cases would expand automation across device, sensor, and app eSource data to/from EHRs, a data broker, and/or technology vendor cloud service to share certified copies with sponsors in near real time. Furthermore, eSource (e.g., DDC) may be used to automatically prompt sites to determine the required protocol assessments and, perhaps more importantly, whether assessments have been already completed.

In the desired future state, a clinical trial participant’s identification number would need to be linked to the clinical trial participant’s medical record number in an eSource modality to ensure data accuracy and seamless interoperability between healthcare and research. This linkage would need to factor in privacy, data integrity, and data traceability considerations.

### Challenges

#### Management of Unstructured Data

*Unstructured data (e.g., Investigator notes/comments) and partial data (e.g., research data not available in EHR) require manual transcription and human intervention*.

*Proposed solution* Use technologies that support conversion of unstructured data. Management of unstructured data requires strong partnership with technology vendors who can help convert the unstructured data to a structured form, using emerging trends like Artificial Intelligence (AI) and Natural Language Processing (NLP). There are opportunities to leverage academia, EHR vendors, and standards to enable organizations to develop and integrate research templates in EHRs (see Data Engineering and Analytics in Fig. 1).

*Value* The ability to use unstructured data and reduce the amount of partial data for research purpose will avoid manual transcription of the data (and associated human error) and improve data traceability.

#### Limited Interoperability Between Healthcare and Clinical Research Systems

*There is limited interoperability between healthcare (e.g., EHR) and clinical research systems and applications (e.g., EDC, data warehouse)*.

*Proposed solution* Sponsors should actively participate in the development, use, and promotion of industry standards for interoperability, collaborate with vendors and sites, and design protocols with healthcare interoperability in mind. TransCelerate and HL7 are collaborating to explore ways to facilitate interoperability (e.g., data mapping, creation of common datasets, HL7 working groups, and participation in FHIR Connectathons) [30].

*Value* Interoperability allows full use of the data generated as part of the clinical workflow at sites. Standardized data exchange from disparate systems reduces the burden on data providers and sponsors (see Data Sources, Acquisition, Data Lineage & Traceability, and Data Engineering in Fig. 1).

#### Inconsistency in the Use of eSource Standards

*Inconsistencies in the outputs provided by various eSource modalities at sites produces inefficiencies in consuming data*.

*Proposed solution* Better support for clinical research, using existing or new eSource standards, would ensure the outputs are consistent, regardless of eSource modality [30].

*Value* Common output standards would increase efficiency and reduce burden on sites, vendors and sponsors (see Data Sources, Acquisition, Data Lineage & Traceability, and Data Engineering in Fig. 1).

#### Limited Scalability of Data Integrations

*Limited ability to scale eSource vendor-data integrations with EDC or data warehouse to store large volumes of data*.

*Proposed solution* Industry, sponsor, and technology vendor collaboration are required to develop data standards, exchange standards, and data transfer agreements in combination with a flexible sponsor system architecture. The current sponsors’ approaches support a more limited use of eSource, which relies heavily on summary data. Some off-the-shelf applications and devices stream raw data, which may not be useful for final analysis; hence, sponsors should consider moving towards an architecture that supports eSource-centric enhancements such as cloud infrastructure, APIs, big data streams from devices, accommodation of large volumes of data, and transformation of raw data into summary data (see Acquisition, Data Engineering, and Data Integration in Fig. 1).

*Value* Building the local, regional, and global infrastructure enables scaled global clinical trials and facilitates near real-time surveillance of data for faster decision-making. It also enables sites and physicians to apply new eSource capabilities to support local needs and new research.

## Key Area III: New Roles

The desired future state will affect the roles and responsibilities for most of the stakeholders. This may require a new skillset, supported by appropriate training on modalities, to support clinical trial conduct. Sponsors and other stakeholders will increase their application of data analytics to enable new clinical trial designs, possibly increasing the number of pragmatic clinical trials and improving clinical trial execution. Additionally, an increase in eSource and informatics acumen across all stakeholders will result in improved and faster data review and analysis techniques, which may have a positive impact on clinical development time and costs, ultimately accelerating the drug development process.

Developing skillsets in applying technology to raw and summarized eSource data will enable faster access and more reliable data, leading to faster generation of insights.

### Challenges

#### Lack of Clarity on Validation and Compliance

*Validation and compliance requirements for the collection of research data are unclear*.

*Proposed solution* Increase training and awareness. More internal training is recommended for personnel to understand validation/regulatory requirements (e.g., Part 11 of Title 21 of the US Code of Federal Regulations [21CFRP11], the EU General Data Protection Regulation [GDPR] [32], and the US Health Insurance Portability and Accountability Act [HIPAA] [33]). Because training is dependent on the modality being used, there needs to be adequate training and awareness within the organization to understand the requirements for the modality being used. For EHR, because sponsors are responsible for assessing the validity, reliability, and integrity of any data used to support a marketing application for a medical product, it is imperative for the sponsor to confirm that the EHR has adequate controls in place to ensure confidentiality, integrity, and reliability of data [5, 6]. Sponsors should ensure vendors and their solutions are qualified and in compliance with appropriate regulations. Sponsors should apply a risk-based approach.

*Value* Risk-avoidance. Knowledge of applicable regulations results in avoidance of risk, related to non-compliance with regulatory requirements (see Adherence to Regulatory Requirements in Fig. 1).

#### Lack of Informatics and eSource Acumen

*Clinical Trial Team Members and Supporting IT Teams do not yet have appropriate eSource and informatics acumen*.

*Proposed Solution* Stakeholder eSource acumen should be increased through a combination of mind set change, training, and strategic hiring ensuring a formation of a cross functional team to advance the discipline. The training should be appropriately tailored to the consumer of the data (e.g., the analytics used by sites and sponsors may be different and require training on appropriate interpretation of the reports). This includes training on emerging trends, such as artificial intelligence, machine learning, big data analytics, and standards (e.g., FHIR). Figure 1 eSource Logical Architecture gives a holistic picture of the current acumen to be expected from an eSource SME. An eSource SME should know the challenges and opportunities for each element and seek guidance from other SMEs if additional details are required.

*Value* New data sources come with possibilities for new and improved clinical insights. A skilled workforce enables faster adoption of the technology and improved transparency of data. Common understanding between inspectors and sponsors would facilitate the inspection and data review process.

## Key Area IV: Regulatory Alignment

With continued dialogue between regulators and research stakeholders, clarity of the regulatory environment concerning eSource implementation will increase. For the future state, stakeholders must align on requirements, expectations, and acceptable eSource approaches. This includes alignment on topics such as privacy, security, data hosting and infrastructure, certified copies, contemporaneous copies and/or access, medical device classification, risk-based computer system validation [18], utilization of an agile validation framework, and acceptable evidence for digital biomarker outcomes to replace traditional gold standard assessments.

Sponsors will ensure (through contractual agreements with third parties providing technology or services and audits) that applicable regulatory requirements are met, and that the distribution of responsibilities is documented [34].

We propose that sponsors seek input from the relevant regulatory agencies whenever a new idea in the eSource domain has had a successful proof of concept. Ideally, this would occur prior to deploying it to scale.

### Challenges

#### Lack of Adaptability in Validation for Technology

*Conventional computer system validation processes do not adapt to the changing landscape of eSource technology needs (e.g., apps, devices, DDC). Validation methods for devices differ from traditional computer systems*.

*Proposed solution* Modernizing computer system validation by adopting agile framework approaches to software development will allow for more flexibility and control over the quality of the final product, quicker development, and better outcomes. Any risk-based or agile validation methods must be GxP compliant [18]. Risk-based methods have not been widely adopted in the industry; thus, additional efforts, such as sharing successful implementations or working with professional societies to promote such methods at conferences and workshops, are needed (see Adherence to Regulatory Requirements in Fig. 1).

*Value* Modern validation processes are needed to keep up with the fast-changing landscape of eSource devices and address the need for more clinical trial participant-centric solutions enabling simpler clinical trial participation.

#### Lack of Adaptability in Validation for EHR Systems

*Global EHR certification is not established*.

*Proposed solution* EHR systems that are certified by the US Office of the National Coordinator for Health Information Technology (ONC) should be exempt from validation requirements [6]. If the EHR is not ONC certified or the ONC certification is not recognized in countries outside the United States, the sponsor will have to ensure that the site has validated the EHR and the proper controls (e.g., access controls, data lineage, audit trails, security, and data protection) are in place (see Adherence to Regulatory Requirements in Fig. 1).

*Value* This could provide considerable time savings and reduce the burden on sponsors and sites, which would not have to validate certified systems. This would also accelerate clinical trial start times. However, these benefits will be truly reached only if a global EHR certification program can be established.

#### Lack of Clarity in Privacy and Security Laws

*Privacy and security laws with respect to eSource are unclear. The ecosystem is different from traditional electronic data capture and data flow*.

*Proposed solution* Specific training should be deployed within the organization to create awareness of the global requirements on data privacy (e.g., the EU GDPR [32], and the current US HIPAA laws [33]) and specific strategies (e.g., creation of standards which do not collect clinical trial participant date of birth [DOB], participant initials) should be implemented to ensure compliance. Systemic audits should be performed for all systems used in data collection and integration to ensure that data security, including access management, is not compromised and data integrity, with appropriate level of encryption (i.e., at transit and at rest) is maintained during data exchange. We should also seek alignment from global regulatory agencies on privacy and security aspects of eSource (see Data Security & Integrity and Adherence to Regulatory Requirements in Fig. 1).

*Value* Diligence in training and auditing will ensure protection of the clinical trial participants’ rights and compliance with regulatory requirements.

## Key Area V: Collaboration

Alignment across stakeholders is key in achieving the eSource future state. Optimizing clinical trial designs, enabling end-to-end data flow, developing and interpreting standards, and equipping the workforce require open communication, knowledge sharing, and cooperation across all parties. A diverse range of skills and expertise will be needed to address the challenges of eSource and bridge the gap between traditional methods and nascent technologies.

### Challenges

#### Lack of Coordinated Industry Efforts

*There is a lack of coordinated efforts from the sponsor community, technology vendors, and other industry forums, to collaborate with global regulatory agencies to create consistent, clear guidance for eSource. This partnership between regulatory agencies and industry is vital to advance eSource adoption*.

*Proposed solution* Industry forums (e.g., TransCelerate, SCDM, eClinical Forum, IMI, and Drug Information Association [DIA]) should collaborate to develop guidelines, tools, standards, eSource adoption templates, data collection methodologies, and best practices for successful eSource end-to-end adoption. They should also engage global regulators and solicit their feedback on these deliverables, working towards developing Global Regulatory Guidance for eSource Implementations. The goal is to ensure that we are collecting the right data in the right way (or the most efficient way) with sufficient precision.

*Value* Aligned approaches on adoption of eSource and best practices facilitate improved clinical research, ultimately accelerating the drug development timeline.

#### Device Mode Equivalency Testing

*Device mode equivalency testing does not meet today’s eSource technology needs for volume, time, and cost (e.g., Bring Your Own Device [BYOD] vs dedicated devices)*.

*Proposed solution* Equivalency could be ascertained through mode-equivalency validation trials by sponsors or consortia. Trustworthy equivalence could be achieved through full transparency and the ability for a wide range of participants to test the validity of the equivalence (see Data Sources and Adherence to Regulatory Requirements in Fig. 1).

*Value* Publishing results for consumption by others in the industry may be an effective way to decrease the need to repeat such equivalency testing [35].

#### Clinical Trial Participant Burden

*Clinical trial participants are burdened by carrying multiple devices*.

*Proposed solution* Utilization of BYOD whenever possible would reduce the device-carrying burden of clinical trial participants. BYOD proposals should consider the following two types of measures:

I. For licensed measures, use of BYOD requires approval by the license holders. A library of instruments approved by license holders should be built and made available in the public domain.
II. For unlicensed measures, sponsors, in collaboration with technology vendors, should confirm that testing has been performed and is compatible with various platform versions. Sponsors should ensure technology vendors include additional measures to verify the accuracy of data (e.g., timestamps). Sponsors should take a risk-based approach (e.g., use for clinical trial participant engagement).

Accommodations, however, should be made for those patients that do not have their own device that would be suitable for use with relevant e-Source technology.

*Value* Clinical trial participant burden is reduced because the participant can utilize one device for both personal use and clinical trial participation. Adherence may be improved when the participants have a choice to utilize their own device. The use of BYOD introduces efficiencies and cost-benefit from reduction in provisioning of devices (see Data Sources, Data Security & Integrity, and Adherence to Regulatory Requirements in Fig. 1).

#### Lack of Classifications and Qualifications

*Medical device/app classifications and digital biomarker qualifications, to be considered a clinical outcome equivalent to a ‘gold standard,’ are nascent (e.g., activity meter data vs six-minute walk test)*.

*Proposed solution* There is an opportunity for professional societies, organizations, and/or vendors to create a library and research knowledgebase that contains qualified devices and documented rationale. A medical device decision tree or tool, developed in partnership with regulators, would be beneficial. The library would also seek to address the following gaps:

- FDA guidance has provided support to device selection and classification [36], but there is still a gray area that is subject to interpretation. Likewise, digital biomarker development information is limited.
- For digital biomarker development, the Clinical Trial Transformation Initiative (CTTI) [37] has developed recommendations and tools that may be helpful for selecting appropriate mobile outcomes as future clinical trial endpoints [38, 39]. Additional regulatory guidance on process expectations in establishing a digital biomarker would provide consistency in approach and expedite research.

*Value* A global platform to facilitate best practices, transparency, and validation of digital biomarkers would accelerate clinical evidence generation and generation of new insights (see Adherence to Regulatory Requirements in Fig. 1).

## Conclusion and Next Steps

Global clinical data collection, analysis, transfer, and use are evolving. Data collection is changing from paper-based sources to electronic sources requiring interoperability, end-to-end traceability, and secure data exchange. This evolution is being supported and facilitated by advancements in eSource standard development processes, improved interoperability, clarity in regulatory guidance, increasing numbers of sites utilizing qualified EHRs and ICH-compliant DDC research systems, and increasing numbers and maturity of nascent devices, applications, and state-of-the-art health technologies, including digital health technologies.

To achieve full adoption of eSource and keep up with the rapid development of technological capabilities, sponsors, sites, clinical trial participants, standard-setting organizations, technology experts, payers, regulators, and industry forums need to come together with a call to action in pursuit of the following:

- Further development of best practices by seeking harmonized regulatory guidance and aligning on compliance with ICH Good Clinical Practice (GCP) and local laws (e.g., sponsor’s responsibilities related to diligence and maintenance of device calibration; site, sponsor, and technology vendor roles in contemporaneous data collection, and eSource data hosting responsibilities regarding data privacy),
- Create and evolve an end-to-end framework (covering processes, technology, metrics, and people) to help facilitate eSource adoption throughout the research enterprise,
- Cultivate an environment where eSource access and ease of use are propitious for clinical trial participants, sites, and sponsors,
- Collaborate with Standards Development Organizations (SDOs), such as HL7, and other industry forums to define use cases, promote interoperability, and use data and exchange standards, and
- Build shared knowledge such that scientific value and improvements to clinical trial data integrity are strengthened by eSource.

We have not covered all perspectives in this paper since the challenges and the value of e-Source may be different based on the wide variety of roles involved in execution of a clinical trial. In addition to the challenges above we need to consider other complexities associated with introduction of e-Source in clinical trials, e.g., bias that may be introduced due to selection of sites/participants which support the implementation of e-Source-based trials. Execution of clinical trials is a complex scientific and operational process and industry has to come together engaging diverse perspectives and be inclusive of different stakeholders including patients.

As the TransCelerate eSource Initiative continues to explore, experiment, and learn, we intend to facilitate this journey to full adoption of eSource for clinical research by.

- Publishing adoption toolkits and best practices from early adopters include the following:
  - Sponsor maturity model,
  - Implementation value calculator,
  - Logical architecture,
  - Enhanced readiness assessment for sites,
  - Roles and responsibilities,
- Continuing to support and engage with multiple stakeholders including SDOs and regulators (e.g., Connectathons, working group meetings),
- Supporting Proof of Concept (PoC) and pilots across the industry and learning from early implementers, and
- Promoting agnostic data interoperability and data exchanges.

eSource is changing the paradigm of clinical research execution. Emerging trends in analytics, improvements in interoperability, and growing support from regulators will enable advancement in adoption of eSource in clinical research. Faster adoption of eSource will accelerate clinical research timelines and improve quality by enabling near real-time access to clinical trial data which will, in turn, enable faster decision-making. Integrity and traceability of the data will improve while reducing the burden on clinical trial participants and clinical trial sites via the minimization of human intervention involved in transcription of the data. This will not only benefit pharmaceutical sponsors, but also clinical research in the academic setting. Regulatory inspections will benefit from increased data availability, directly from the data source. These factors will ultimately help patients improve their quality of life by bringing quality therapies and devices to the market faster and reducing site and clinical trial participant burdens.
